# Supplementary material for: Lipid-induced transcriptomic changes in blood link to lipid metabolism and allergic response
Source: Nat Commun. 2023 Feb 1;14:544. doi: 10.1038/s41467-022-35663-x (PMC9892529; doi:10.1038/s41467-022-35663-x)
Supplement: Supplementary file 1 — Supplementary Information [file 41467_2022_35663_MOESM1_ESM.pdf]

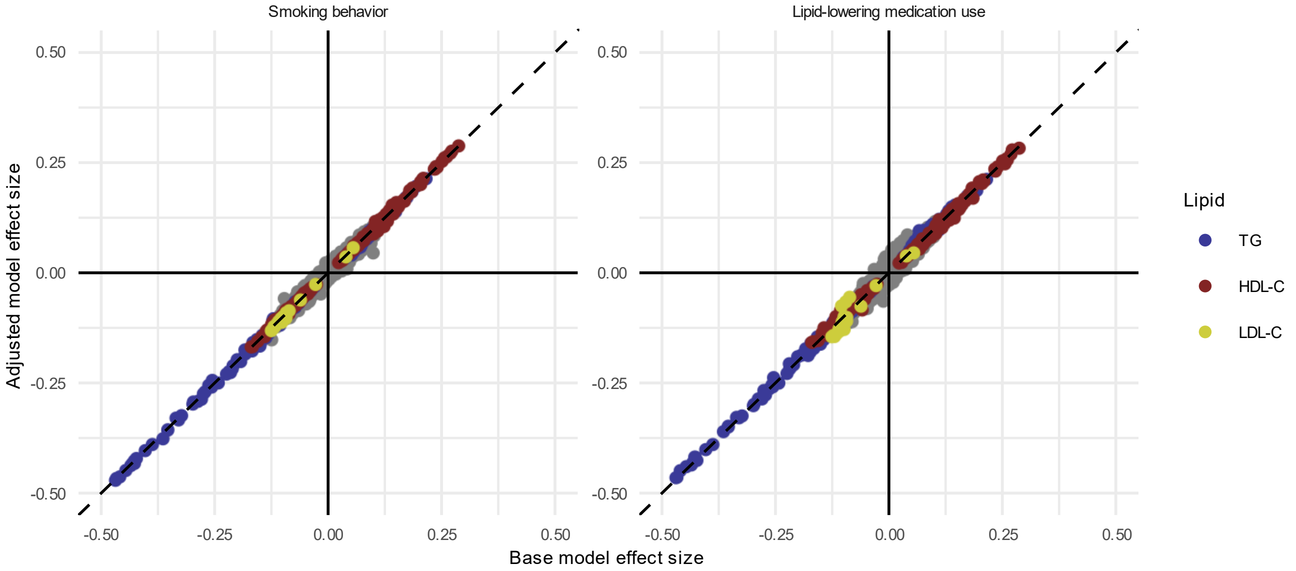

Supplementary Figure 1. Transcriptome-wide analysis effect sizes are not sensitive to additional adjustment for known smoking behavior and lipid-lowering medication use. Linear regression beta effect size in standard deviation per standard deviation with 95% confidence interval. Points depicted in color represent genome-wide significant associations in the model without adjustment for smoking behavior and lipid-lowering medication use.

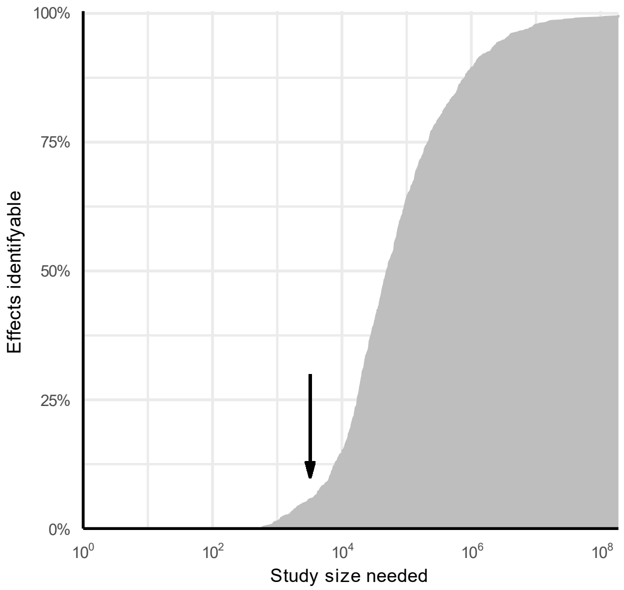

Supplementary Figure 2. Power analysis. Arrow depicts study size of this study.

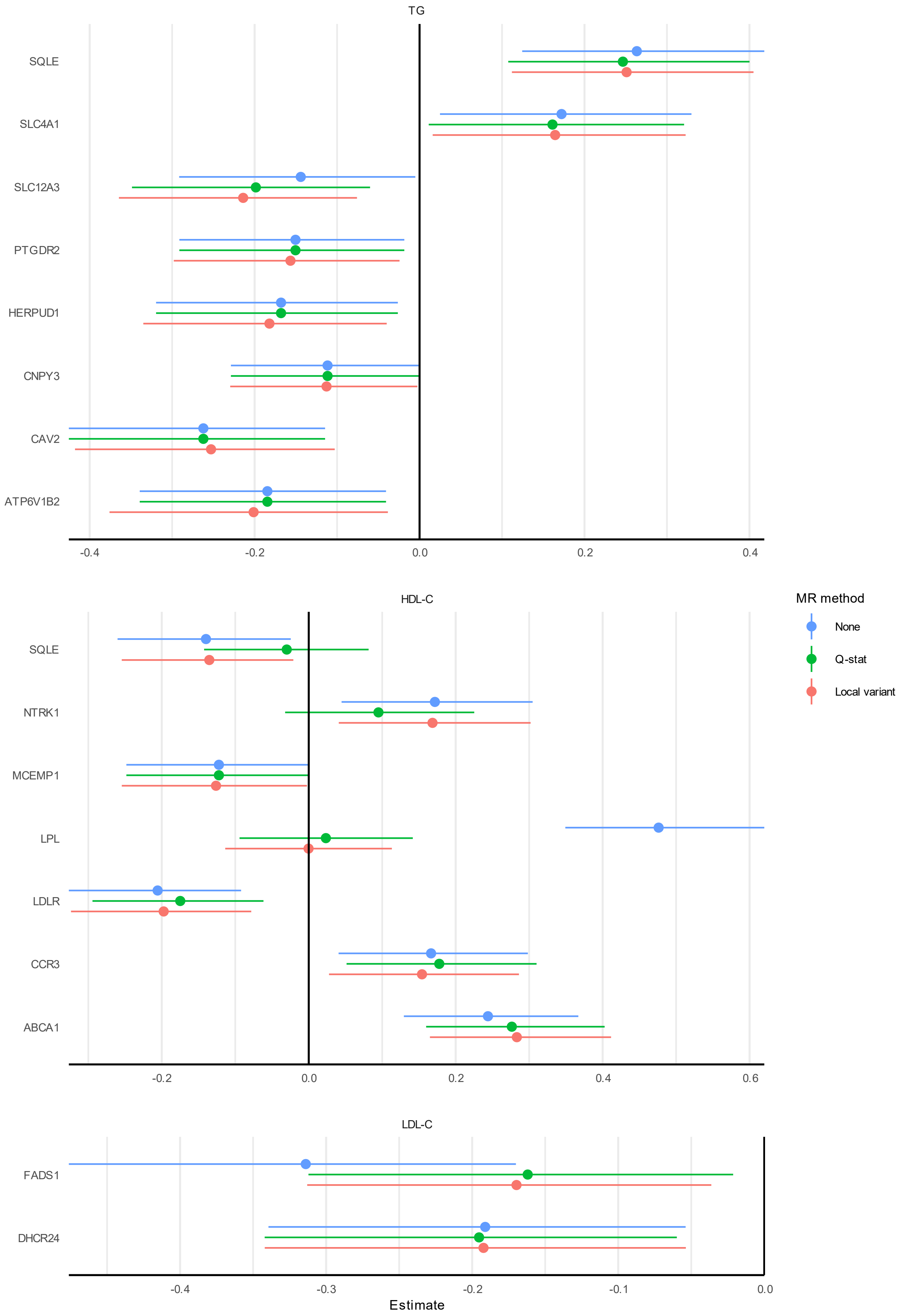

TG
SQLE
SLC4A1
SLC12A3
PTGDR2
HERPUD1
CNPY3
CAV2
ATP6V1B2
-0.4
-0.2
0.0
0.2
0.4
HDL-C
SQLE
NTRK1
MCEMP1
LPL
LDLR
CCR3
ABCA1
-0.2
0.0
0.2
0.4
0.6
LDL-C
FADS1
DHCR24
-0.4
-0.3
-0.2
-0.1
0.0
Estimate
MR method
None
Q-stat
Local variant

Supplementary Figure 3. Cochran's method performs similarly as adjusting for *cis*-expression QTL. Points represent the Wald ratio effect size in standard deviation per standard deviation with 95% confidence interval (n = 3229). Label "None" is the MR estimate without adjustment for pleiotropic effects, label "Q-stat" is the MR estimate adjusted for pleiotropic effects based on Cochran's method, label "Local variant" is the MR estimate adjusted for direct pleiotropic effects by adding the dosage of the *cis*-expression QTL as covariate in the model.

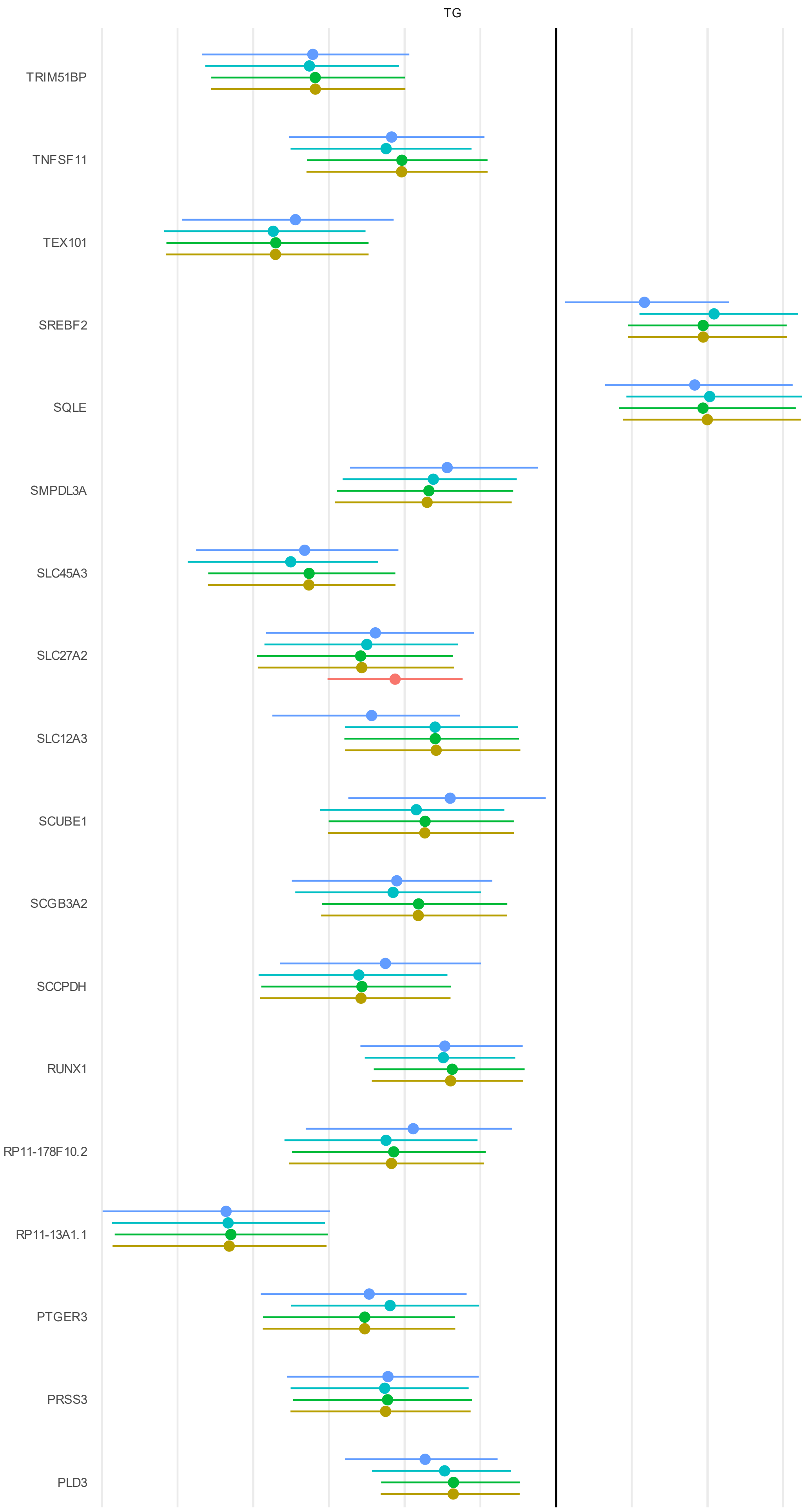
TG
TRIM51BP
TNFSF11
TEX101
SREBF2
SQLE
SMPDL3A
SLC45A3
SLC27A2
SLC12A3
SCUBE1
SCGB3A2
SCCPDH
RUNX1
RP11-178F10.2
RP11-13A1.1
PTGER3
PRSS3
PLD3

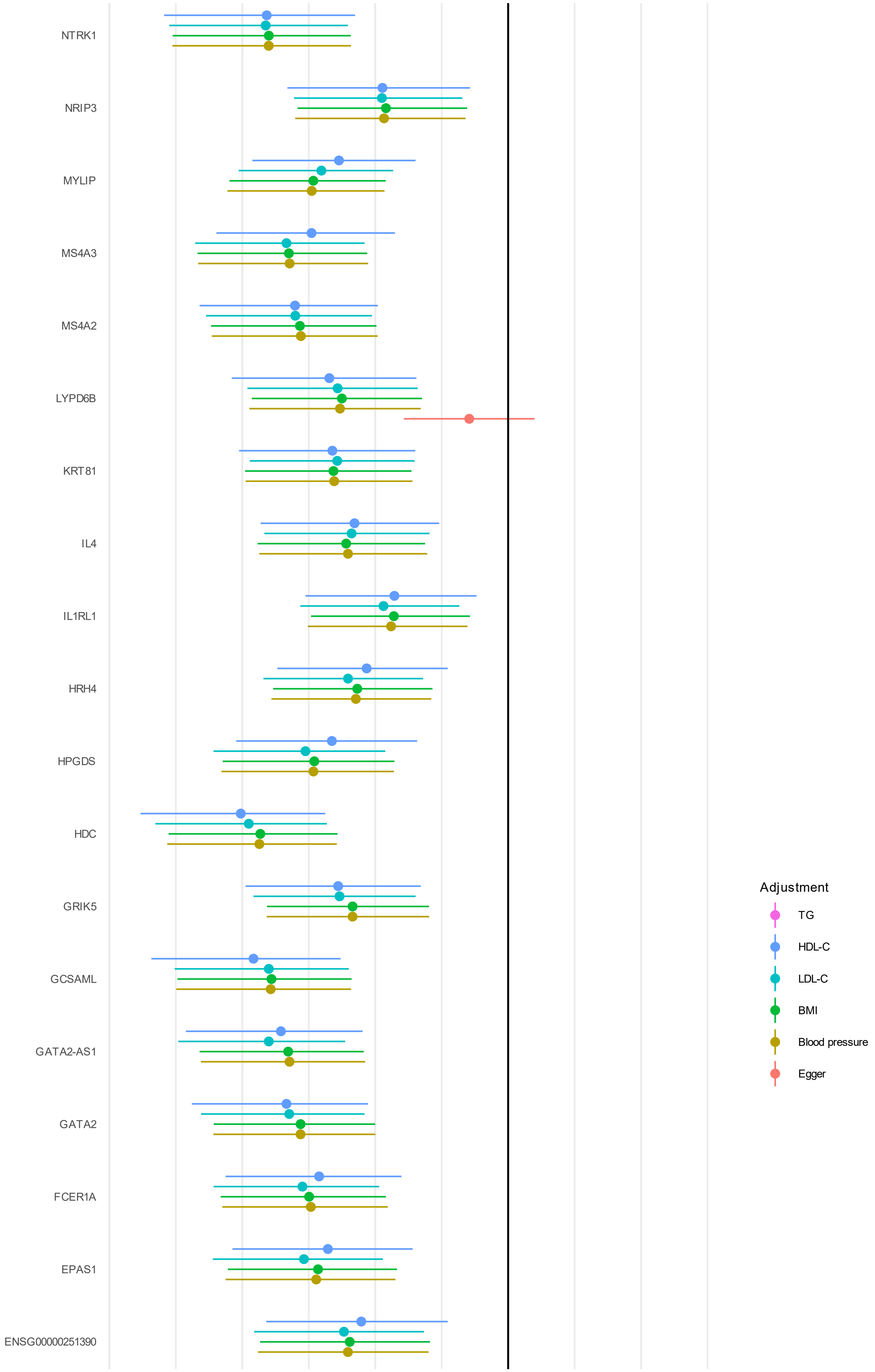
NTRK1
NRIP3
MYLIP
MS4A3
MS4A2
LYPD6B
KRT81
IL4
IL1RL1
HRH4
HPGDS
HDC
GRIK5
GCSAML
GATA2-AS1
GATA2
FCER1A
EPAS1
ENSG00000251390
Adjustment
TG
HDL-C
LDL-C
BMI
Blood pressure
Egger

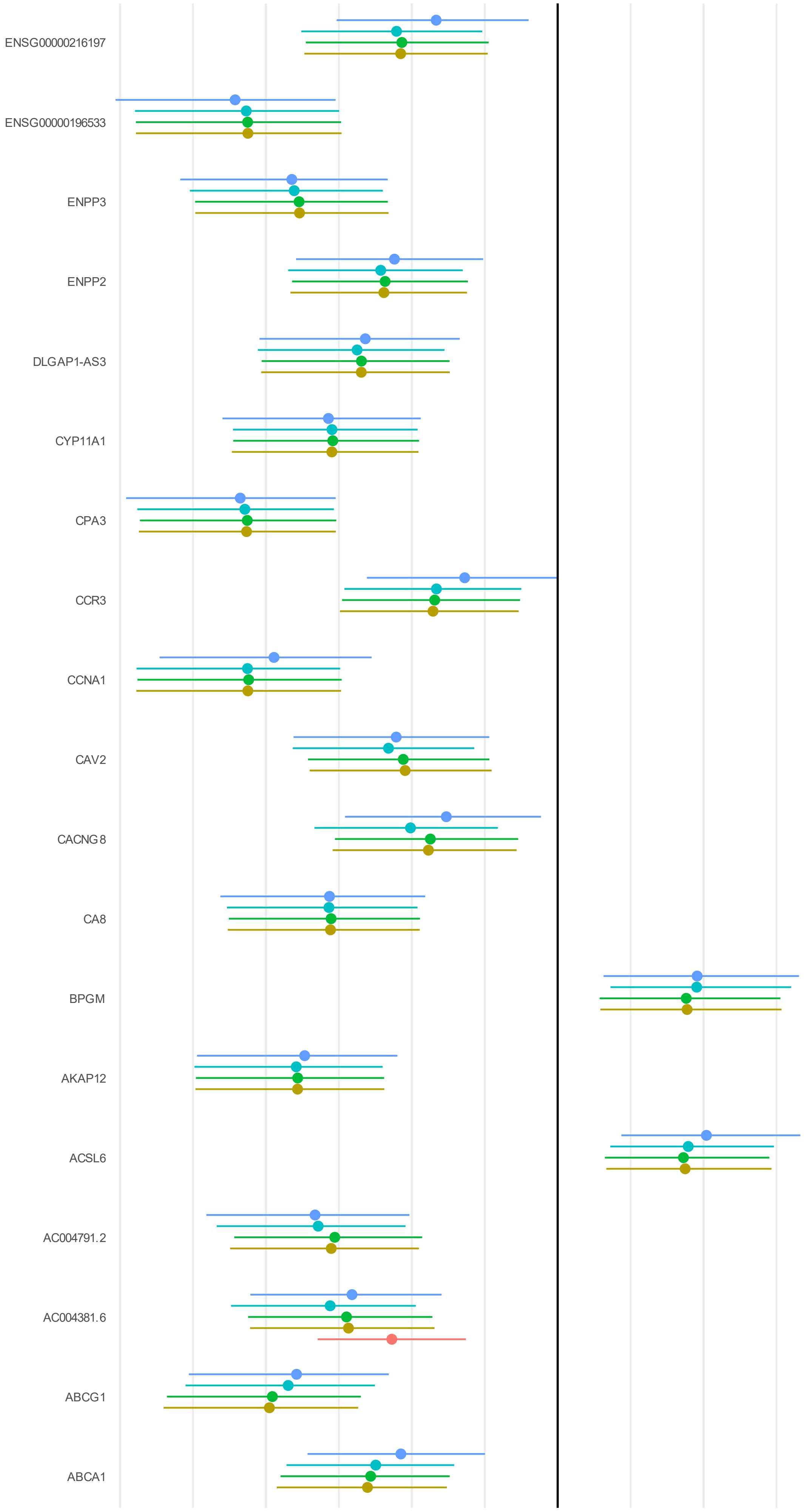
ENSG00000216197
ENSG00000196533
ENPP3
ENPP2
DLGAP1-AS3
CYP11A1
CPA3
CCR3
CCNA1
CAV2
CACNG8
CA8
BPGM
AKAP12
ACSL6
AC004791.2
AC004381.6
ABCG1
ABCA1

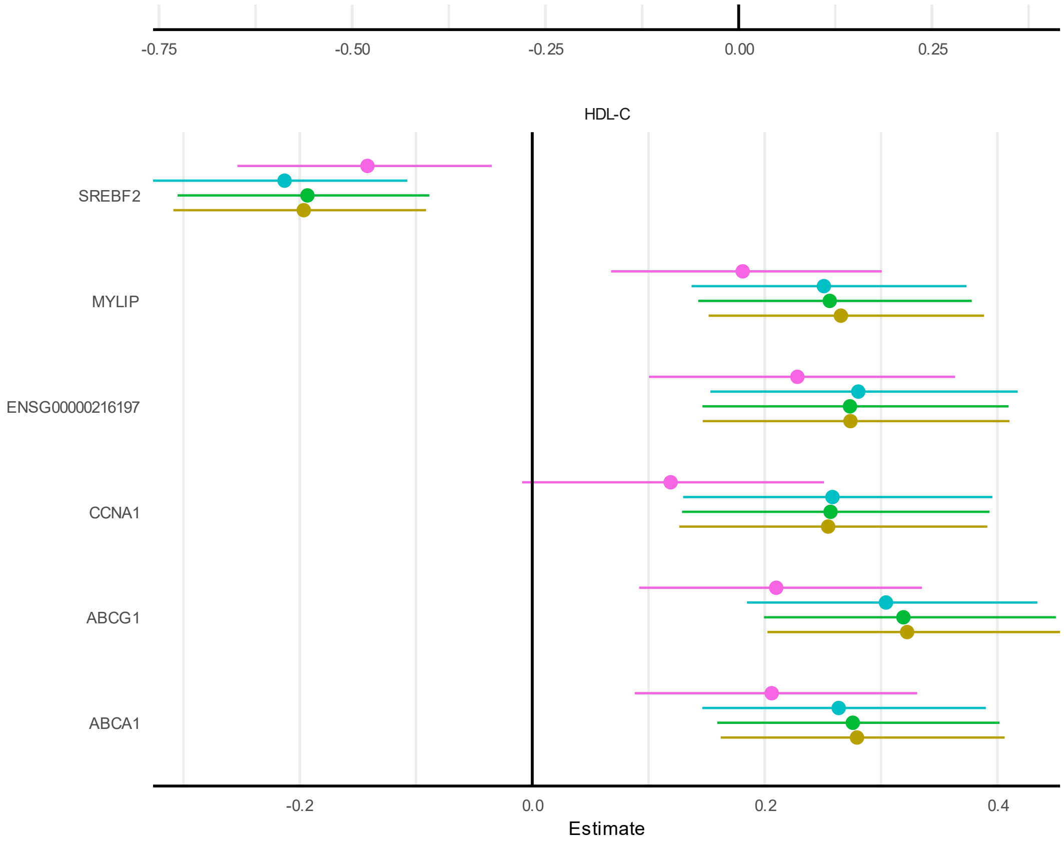

Supplementary Figure 4. MR effect sizes are generally not sensitive to additional adjustment for genetic instruments for the other lipids or genetic instruments for BMI, systolic blood pressure and diastolic blood pressure, or for adjustment using Egger regression. Points represent the Wald ratio effect size in standard deviation per standard deviation with 95% confidence interval (n = 3229).

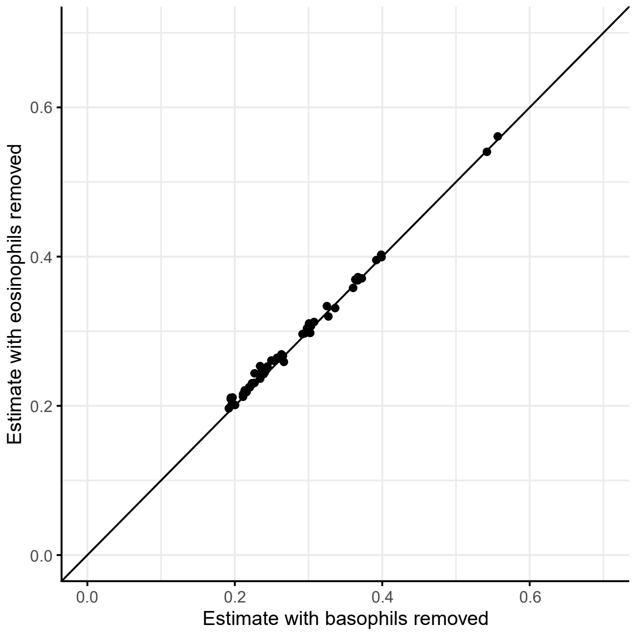

Supplementary Figure 5. Scatter plot of MR estimates with basophils removed and with eosinophils removed from the model. Wald ratio effect size in standard deviation per standard deviation.

BIOS consortium

Management Team
Bastiaan T. Heijmans (chair)[1], Peter A.C. ’t Hoen[2], Joyce van Meurs[3], Rick Jansen[5], Lude Franke[6].

Cohort collection
Dorret I. Boomsma[7], René Pool[7], Jenny van Dongen[7], Jouke J. Hottenga[7] (Netherlands Twin Register); Marleen MJ van Greevenbroek[8], Coen D.A. Stehouwer[8], Carla J.H. van der Kallen[8], Casper G. Schalkwijk[8] (Cohort study on Diabetes and Atherosclerosis Maastricht); Cisca Wijmenga[6], Lude Franke[6], Sasha Zhernakova[6], Ettje F. Tigchelaar[6] (LifeLines Deep); P. Eline Slagboom[1], Marian Beekman[1], Joris Deelen[1], Diana van Heemst[9] (Leiden Longevity Study); Jan H. Veldink[10], Leonard H. van den Berg[10] (Prospective ALS Study Netherlands); Cornelia M. van Duijn[4], Bert A. Hofman[11], Aaron Isaacs[4], André G. Uitterlinden[3] (Rotterdam Study).

Data Generation
Joyce van Meurs (Chair)[3], P. Mila Jhamai[3], Michael Verbiest[3], H. Eka D. Suchiman[1], Marijn Verkerk[3], Ruud van der Breggen[1], Jeroen van Rooij[3], Nico Lakenberg[1].

Data management and computational infrastructure
Hailiang Mei (Chair)[12], Maarten van Iterson[1], Michiel van Galen[2], Jan Bot[13], Dasha V. Zhernakova[6], Rick Jansen[5], Peter van ’t Hof[12], Patrick Deelen[6], Irene Nooren[13], Peter A.C. ’t Hoen[2], Bastiaan T. Heijmans[1], Matthijs Moed[1].

Data Analysis Group
Lude Franke (Co-Chair)[6], Martijn Vermaat[2], Dasha V. Zhernakova[6], René Luijk[1], Marc Jan Bonder[6], Maarten van Iterson[1], Patrick Deelen[6], Freerk van Dijk[14], Michiel van Galen[2], Wibowo Arindrarto[12], Szymon M. Kielbasa[15], Morris A. Swertz[14], Erik. W van Zwet[15,] Rick Jansen[5], Peter-Bram ’t Hoen (Co-Chair)[2], Bastiaan T. Heijmans (Co-Chair)[1].

1. Molecular Epidemiology, Department of Biomedical Data Sciences, Leiden University Medical Center, Leiden, The Netherlands
2. Department of Human Genetics, Leiden University Medical Center, Leiden, The Netherlands
3. Department of Internal Medicine, ErasmusMC, Rotterdam, The Netherlands
4. Department of Genetic Epidemiology, ErasmusMC, Rotterdam, The Netherlands
5. Department of Psychiatry, VU University Medical Center, Neuroscience Campus Amsterdam, Amsterdam, The Netherlands
6. Department of Genetics, University of Groningen, University Medical Centre Groningen, Groningen, The Netherlands
7. Department of Biological Psychology, VU University Amsterdam, Neuroscience Campus Amsterdam, Amsterdam, The Netherlands
8. Department of Internal Medicine and School for Cardiovascular Diseases (CARIM), Maastricht University Medical Center, Maastricht, The Netherlands
9. Department of Gerontology and Geriatrics, Leiden University Medical Center, Leiden, The Netherlands
10. Department of Neurology, Brain Center Rudolf Magnus, University Medical Center Utrecht, Utrecht, The Netherlands
11. Department of Epidemiology, ErasmusMC, Rotterdam, The Netherlands
12. Sequence Analysis Support Core, Department of Biomedical Data Sciences, Leiden University Medical Center, Leiden, The Netherlands
13. SURFsara, Amsterdam, the Netherlands
14. Genomics Coordination Center, University Medical Center Groningen, University of Groningen, Groningen, the Netherlands
15. Medical Statistics, Department of Biomedical Data Sciences, Leiden University Medical Center, Leiden, The Netherlands
