# Supplementary material for: Lipid-induced transcriptomic changes in blood link to lipid metabolism and allergic response
Source: Nat Commun. 2023 Feb 1;14:544. doi: 10.1038/s41467-022-35663-x (PMC9892529; doi:10.1038/s41467-022-35663-x)
Supplement: Supplementary file 2 — Description of Additional Supplementary Files [file 41467_2022_35663_MOESM2_ESM.pdf]

Description of Additional Supplementary Files

File Name: Supplementary Data 1
Description: **Results of the transcriptome-wide analysis on lipid levels.** Presented are genome-wide significant linear regression results after adjusting for 17,740 tests, i.e. the number of genes, using the Bonferroni method. Effect size in standard deviation per standard deviation. Column Direction contains the direction of association for cohorts CODAM, LL, LLS, NTR and PAN, respectively.

File Name: Supplementary Data 2
Description: **Genetic variants used to create genetic instruments.** Variants with P-value < 5×10^{-8} that were >1 Mb apart and nearly independent ($r^2 < 0.10$) reported in a GWAS on lipids (17).

File Name: Supplementary Data 3
Description: **Strength of genetic instruments.** Linear regression effect size in standard deviation per standard deviation. Presented are the nominal P-values.

File Name: Supplementary Data 4
Description: **Associations between genetic instruments and potential confounders.** Presented are the nominal P-values of the linear regression model.

File Name: Supplementary Data 5
Description: **Results of the forward MR analysis: blood lipids affect transcription.** Presented are significant results after adjusting for 496 (TG), 284 (HDL-C) and 26 (LDL-C) tests using the Benjamini-Hochberg method at 5% FDR. Wald ratio effect size in standard deviation per standard deviation. Column Removed.rsID describes the genetic variants removed from the analysis due to pleiotropy. Column Q-stat shows the Q-statistic P-value after removing potential pleiotropic variants. Column N.power is the number of samples required to detect a causal effect at Power = 0.8. Column Pleiotropy shows what pleiotropic effects were detected. Column Pleiotropy describes if and at what stage an effect was excluded based on several pleiotropy sensitivity analyses.

File Name: Supplementary Data 6
Description: **Results of the reverse MR analysis: transcription does not affect blood lipid levels.** Presented are results for the 56 genes affected by TG and the 6 genes affected by HDL-C in the forward MR analysis, see Supplementary Data 5. Wald ratio effect size in standard deviation per standard deviation. P-values corrected for multiple testing using the Benjamini-Hochberg method at 5% FDR. For genes *BPGM* and *RUNX1* no eQTLs were found at 5% FDR (18).

File Name: Supplementary Data 7
Description: **Overlap between lipid-affected genes found in this study and TWAS and MR genes found in earlier studies**
Column Bartel and Inouye refer to the lipid-associated genes found in earlier TWAS (13,14). Column Porcu refers to the lipid-affected genes found in an earlier two-sample MR study (23).

File Name: Supplementary Data 8

Description**: Pathway enrichment analysis shows TG-affected genes have a function in lipid metabolism and allergy.** Pathway enrichments generated using a one-sided Fisher's exact test with clusterProfiler (56) using the 10 human pathway databases BioPlanet 2019, WikiPathways 2019 Human, KEGG 2019 Human, Elsevier Pathway Collection, BioCarta 2015, Reactome 2016, HumanCyc 2016, NCI-Nature 2016, Panther 2016 and MSigDB Hallmark 2020. P-values corrected for multiple testing using the Benjamini-Hochberg method at 5% FDR over all databases.

File Name: Supplementary Data 9

Description: **Several TG-affected genes have a causal role in allergic diseases.** Wald ratio P-values corrected for multiple testing using the Benjamini-Hochberg method at 5% FDR.
