# Supplementary material for: Lipid-induced transcriptomic changes in blood link to lipid metabolism and allergic response
Source: Nat Commun. 2023 Feb 1;14:544. doi: 10.1038/s41467-022-35663-x (PMC9892529; doi:10.1038/s41467-022-35663-x)
Supplement: Supplementary file 12 — Reporting Summary [file 41467_2022_35663_MOESM12_ESM.pdf]

Corresponding author(s): Bastiaan T. Heijmans
Last updated by author(s): Oct 30, 2022

Reporting Summary

Nature Portfolio wishes to improve the reproducibility of the work that we publish. This form provides structure for consistency and transparency in reporting. For further information on Nature Portfolio policies, see our Editorial Policies and the Editorial Policy Checklist.

Statistics

For all statistical analyses, confirm that the following items are present in the figure legend, table legend, main text, or Methods section.

n/a	Confirmed	
☐	☒	The exact sample size (*n*) for each experimental group/condition, given as a discrete number and unit of measurement
☐	☒	A statement on whether measurements were taken from distinct samples or whether the same sample was measured repeatedly
☐	☒	The statistical test(s) used AND whether they are one- or two-sided<br>*Only common tests should be described solely by name; describe more complex techniques in the Methods section.*
☐	☒	A description of all covariates tested
☐	☒	A description of any assumptions or corrections, such as tests of normality and adjustment for multiple comparisons
☐	☒	A full description of the statistical parameters including central tendency (e.g. means) or other basic estimates (e.g. regression coefficient) AND variation (e.g. standard deviation) or associated estimates of uncertainty (e.g. confidence intervals)
☐	☒	For null hypothesis testing, the test statistic (e.g. *F*, *t*, *r*) with confidence intervals, effect sizes, degrees of freedom and *P* value noted<br>*Give P values as exact values whenever suitable.*
☒	☐	For Bayesian analysis, information on the choice of priors and Markov chain Monte Carlo settings
☐	☒	For hierarchical and complex designs, identification of the appropriate level for tests and full reporting of outcomes
☐	☒	Estimates of effect sizes (e.g. Cohen's *d*, Pearson's *r*), indicating how they were calculated

Our web collection on statistics for biologists contains articles on many of the points above.

Software and code

Policy information about availability of computer code

Data collection	R-code used to preprocess the data is available at: https://github.com/bbmri-nl/BBMRIomics.<br>Software: FastQC v0.10.1, cutadapt v1.1, Sickle v1.2, STAR v2.3.0e, R v4.0.3, edgeR v3.28.1
Data analysis	R-code used to perform the analyses is available at: www.github.com/kfdekkers/twasmr.<br>Software: R v4.0.3, v4.2.0, cate v1.1.1, bacon v1.14.0, clusterProfiler v4.4.0

For manuscripts utilizing custom algorithms or software that are central to the research but not yet described in published literature, software must be made available to editors and reviewers. We strongly encourage code deposition in a community repository (e.g. GitHub). See the Nature Portfolio guidelines for submitting code & software for further information.

Data

Policy information about availability of data

All manuscripts must include a data availability statement. This statement should provide the following information, where applicable:

- Accession codes, unique identifiers, or web links for publicly available datasets
- A description of any restrictions on data availability
- For clinical datasets or third party data, please ensure that the statement adheres to our policy

The RNA sequencing data generated in this study and phenotypes age, sex and cell types have been deposited in the EGA database under accession code EGAS00001001077 [https://ega-archive.org/studies/EGAS00001001077/], and data access procedures are available at https://www.bbmri.nl/acquisition-use-

analyze/bios. The genotype and other phenotypes, data are governed by the respective biobanks. Access can be requested according to the procedures established by the biobanks, with restrictions imposed by the respective institutional review boards and Dutch law. The Supplementary Figures generated in this study are provided in the Supplementary Information file and the Supplementary Tables are provided as separate Supplementary Data files. Public databases used in this study include human genome (build NCBI37, https://www.ncbi.nlm.nih.gov/assembly/GCF_000001405.13/), Ensembl v.71 (https://www.ensembl.info/2013/04/11/ensembl-71-has-been-released/), and pathway databases BioPlanet 2019, WikiPathways 2019 Human, KEGG 2019 Human, Elsevier Pathway Collection, BioCarta 2015, Reactome 2016, HumanCyc 2016, NCI-Nature 2016, Panther 2016 and MSigDB Hallmark 2020 downloaded from https://maayanlab.cloud/Enrichr/#libraries. Public datasets used in this study include those from Willer et al. (17), Võsa et al. (18), Locke et al. (21), Warren et al. (22), Monaco et al. (24), Ferreira et al. (25,26), Nikpay et al. (28) and Okada et al (29).

17. Willer CJ, Schmidt EM, Sengupta S, Peloso GM, Gustafsson S, Kanoni S, et al. Discovery and refinement of loci associated with lipid levels. Nat Genet. 2013 Nov;45(11):1274–83.
18. Võsa U, Claringbould A, Westra HJ, Bonder MJ, Deelen P, Zeng B, et al. Large-scale cis- and trans-eQTL analyses identify thousands of genetic loci and polygenic scores that regulate blood gene expression. Nat Genet. 2021 Sep;53(9):1300–10.
21. Locke AE, Kahali B, Berndt SI, Justice AE, Pers TH, Day FR, et al. Genetic studies of body mass index yield new insights for obesity biology. Nature. 2015 Feb;518(7538):197–206.
22. Warren HR, Evangelou E, Cabrera CP, Gao H, Ren M, Mifsud B, et al. Genome-wide association analysis identifies novel blood pressure loci and offers biological insights into cardiovascular risk. Nat Genet. 2017 Mar;49(3):403–15.
24. Monaco G, Lee B, Xu W, Mustafah S, Hwang YY, Carré C, et al. RNA-Seq Signatures Normalized by mRNA Abundance Allow Absolute Deconvolution of Human Immune Cell Types. Cell Rep. 2019 Feb 5;26(6):1627-1640.e7.
25. Ferreira MAR, Mathur R, Vonk JM, Szwajda A, Brumpton B, Granell R, et al. Genetic Architectures of Childhood- and Adult-Onset Asthma Are Partly Distinct. Am J Hum Genet. 2019 Apr 4;104(4):665–84.
26. Ferreira MA, Vonk JM, Baurecht H, Marenholz I, Tian C, Hoffman JD, et al. Shared genetic origin of asthma, hay fever and eczema elucidates allergic disease biology. Nat Genet. 2017 Dec;49(12):1752–7.
28. Nikpay M, Goel A, Won HH, Hall LM, Willenborg C, Kanoni S, et al. A comprehensive 1000 Genomes–based genome-wide association meta-analysis of coronary artery disease. Nat Genet. 2015 Oct;47(10):1121–30.
29. Okada Y, Wu D, Trynka G, Raj T, Terao C, Ikari K, et al. Genetics of rheumatoid arthritis contributes to biology and drug discovery. Nature. 2014 Feb;506(7488):376–81.

Human research participants

Policy information about studies involving human research participants and Sex and Gender in Research.

Reporting on sex and gender

We have used sex as a covariate in the analyses to improve the precision of the estimates. Sex was self-reported and confirmed using genotyping. There was no hypothesis to assume sex differences in our study, and the study was underpowered to perform sex specific analyses.

Population characteristics

See Table 1.

Recruitment

See the following publications of each participating center:

Greevenbroek MMJ van, Jacobs M, Kallen CJH van der, Vermeulen VMMJ, Jansen EHJM, Schalkwijk CG, et al. The cross-sectional association between insulin resistance and circulating complement C3 is partly explained by plasma alanine aminotransferase, independent of central obesity and general inflammation (the CODAM study). Eur J Clin Invest. 2011;41(4):372–9.

Tigchelaar EF, Zhernakova A, Dekens JAM, Hermes G, Baranska A, Mujagic Z, et al. Cohort profile: LifeLines DEEP, a prospective, general population cohort study in the northern Netherlands: study design and baseline characteristics. BMJ Open. 2015 Aug 1;5(8):e006772.

Schoenmaker M, Craen AJM de, Meijer PHEM de, Beekman M, Blauw GJ, Slagboom PE, et al. Evidence of genetic enrichment for exceptional survival using a family approach: the Leiden Longevity Study. Eur J Hum Genet. 2006 Jan;14(1):79–84.

Willemsen G, Vink JM, Abdellaoui A, Braber A den, Beek JHDA van, Draisma HHM, et al. The Adult Netherlands Twin Register: Twenty-Five Years of Survey and Biological Data Collection. Twin Res Hum Genet. 2013 Feb;16(1):271–81.
Ikram MA, Brusselle G, Ghanbari M, Goedegebure A, Ikram MK, Kavousi M, et al. Objectives, design and main findings until 2020 from the Rotterdam Study. Eur J Epidemiol. 2020 May 1;35(5):483–517.

Huisman MHB, Jong SW de, Doormaal PTC van, Weinreich SS, Schelhaas HJ, Kooi AJ van der, et al. Population based epidemiology of amyotrophic lateral sclerosis using capture–recapture methodology. J Neurol Neurosurg Psychiatry. 2011 Oct 1;82(10):1165–70.

Ethics oversight

The study was approved by the institutional review boards of the participating centers and all participants have given written informed consent and the experimental methods comply with the Helsinki Declaration.

Note that full information on the approval of the study protocol must also be provided in the manuscript.

Field-specific reporting

Please select the one below that is the best fit for your research. If you are not sure, read the appropriate sections before making your selection.

☒ Life sciences ☐ Behavioural & social sciences ☐ Ecological, evolutionary & environmental sciences

Life sciences study design

All studies must disclose on these points even when the disclosure is negative.

Sample size	We performed the analyses in all samples of the 6 cohorts of the BIOS consortium with RNA-seq and genotype data, which was not specifically setup to address our research question. We also performed post-hoc power calculations for the Mendelian randomization part, which showed that our analysis was expected to find just 6% of the effects (we found 7%). To find 90% of the effects a sample size of 1 million is needed.
Data exclusions	A detailed description of data generation and preprocessing can be found in Zhernakova et al. Nat Genet 2017. For this study we excluded genes with zero reads in at least 20% of the samples.<br><br>Zhernakova DV, Deelen P, Vermaat M, van Iterson M, van Galen M, Arindrarto W, et al. Identification of context-dependent expression quantitative trait loci in whole blood. Nat Genet. 2017 Jan;49(1):139–45.
Replication	We performed a meta-analysis and triangulation, without explicit replication.
Randomization	All samples were randomized before measurement.
Blinding	We did not perform any group-based analyses.

Reporting for specific materials, systems and methods

We require information from authors about some types of materials, experimental systems and methods used in many studies. Here, indicate whether each material, system or method listed is relevant to your study. If you are not sure if a list item applies to your research, read the appropriate section before selecting a response.

Materials & experimental systems

n/a	Involved in the study	
☒	☐	Antibodies
☒	☐	Eukaryotic cell lines
☒	☐	Palaeontology and archaeology
☒	☐	Animals and other organisms
☒	☐	Clinical data
☒	☐	Dual use research of concern

Methods

n/a	Involved in the study	
☒	☐	ChIP-seq
☒	☐	Flow cytometry
☒	☐	MRI-based neuroimaging
